# Screening Intimate Partner Violence During Pregnancy in Maternity Clinics—Views of Finnish Public Health Nurses

**DOI:** 10.1111/scs.70090

**Published:** 2025-07-25

**Authors:** Elina Lähteenmäki, Helena Leino‐Kilpi, Kaisa Mishina

**Affiliations:** ^1^ Department of Nursing Science University of Turku Turku Finland; ^2^ Turku University Hospital Turku Finland; ^3^ Department of Child Psychiatry University of Turku Finland; ^4^ INVEST Flagship University of Turku Finland

**Keywords:** focus group, intimate partner violence, maternity clinic, pregnancy, screening

## Abstract

**Background:**

Intimate partner violence (IPV) is one of the most common forms of violence against women, occurring across social classes and religions worldwide. Globally, nearly one in three women have experienced IPV in their lifetime. During pregnancy, prevalence rates range from 2.0% to 13.5%.

**Aim:**

This study aimed to analyse the views of public health nurses (PHN) on screening for IPV in Finnish maternity clinics.

**Methods:**

A descriptive study with focus group discussions (*n* = 12 PHNs) and inductive content analysis.

**Results:**

Public health nurses acknowledged that screening for intimate partner violence is part of their responsibilities, even when it is challenging. Barriers to screening included lack of privacy and lack of a common language. PHNs also described difficulties when pregnant women were accompanied by their partners or others during clinic visits, as this limited the possibility for confidential conversations.

**Discussion:**

Public health nurses expressed concern that IPV is often unreported, even when systematic screening is conducted. Many nurses also encountered cultural barriers when addressing IPV. This highlights the need for multilingual screening tools and clear guidelines on how to navigate situations where language barriers or the lack of privacy make communication difficult.

**Conclusion:**

More sensitive and culturally appropriate strategies are needed for the screening of IPV in maternity clinics.

## 1 Introduction

Intimate partner violence (IPV) is one of the most common forms of violence against women, occurring globally in all social classes and cultural contexts [1]. Almost one in three women experiences IPV in their lifetime [1], and the numbers for women during pregnancy range between 2.0% and 13.5% [2]. IPV refers to physical, sexual and psychological harm such as psychological abuse, sexual coercion, or controlling behaviour and is most commonly directed at women, and it is usually inflicted by a current or former male partner [1]. A recent cross‐cultural study including 50 countries reported the highest rates of IPV during pregnancy in Africa and the lowest in Europe, and that the most common forms of IPV during pregnancy are sexual violence, psychological violence, and physical violence [3]. The Nordic countries, known for being the most gender‐equal region in the world, report the highest rates of IPV against women among European nations [4]. In Finland, 34% of women have experienced physical violence or threats of physical violence in their relationships at some point in their lives, and 49% of women have experienced psychological violence [5]. In 2015, Finland committed to the Istanbul Convention to prevent and combat violence against women and domestic violence [6].

IPV during pregnancy is linked to several adversities such as miscarriage, stillbirth, preterm birth, low birth weight [2, 7], postpartum depression, and maternal or newborn death [8]. The most common risk factors for IPV during pregnancy include low education, experience of violence in childhood, inequality in a relationship, attitudes accepting inequality [9], illicit substance use, violence before pregnancy, unmarriedness, low socioeconomic status and unintended pregnancy [10]. Pregnant women can be especially vulnerable due to physical and emotional changes as well as changes in their social network and financial situation [11].

Violence in childhood increases the risk of being in a violent relationship in adulthood [1]. Studies have shown that exposure to IPV is a risk factor for physical or psychological violence against children [12, 13]. Discussions of previous trauma during the perinatal period should occur within a trusting relationship, with support for both pregnant women and health professionals, and access to appropriate follow‐up resources [14]. To break this generational trauma, identifying IPV and providing help for families are critical.

The purpose of IPV screening in healthcare is to identify individuals who have experienced IPV, whether recently or in the past, to offer them support, information, and guidance if needed. In this study, screening refers to asking the client directly or indirectly, using a form or e‐form, about experiences of IPV [15]. Screening for IPV during pregnancy has been studied worldwide [15] from the perspectives of health professionals [16] and victims [17]. IPV is mainly studied by assessing various interventions [18] such as training healthcare professionals to screen for IPV [19]. However, there still is a need for research evidence on how screening takes place when a professional has instructions for standard screening. It seems that victims are not always willing to disclose if they have experienced IPV, even when routinely asked about it [20]. Studies show that screening programmes with a clear structure and guidelines on help and services enable women to report their experienced IPV [21]. On the other hand, only a small percentage of IPV victims report the violence to healthcare professionals [22]. Even if a victim does not want to report their experienced IPV, simply talking about it can help them recognise their situation [23].

IPV is not always standardly screened for among pregnant women [24, 25]. There are clear guidelines for how to screen for IPV in Finnish maternity clinics, but studies show that only a fraction of violence cases are reported in Finland [23]. More understanding of the factors and challenges involved in screening for IPV is needed in healthcare. This study explores the perceptions that public health nurses (PHNs) have of the barriers and facilitators of screening for IPV among pregnant women and their partners in public health maternity clinics. Our aim was to obtain research‐based knowledge that can be used when educating PHNs in maternity clinics and in strengthening screening methods to better achieve equitable quality of care for all. Considering the harmfulness of IPV to the health of both the mother and the fetus, it is important to identify and screen for IPV during pregnancy [2].

## 2 Materials and Methods

### 2.1 Study Design

We followed a descriptive study design with focus group discussions and inductive content analysis [26] to gain in‐depth information. The study did not use an existing framework to investigate IPV; the approach was explorative and inductive. We used focus group discussions to utilise group dynamics and dialogue between the participants and the researcher. We chose to employ discussions instead of interviews so that there would be as much interaction as possible between participants. Moreover, since knowledge on this topic is limited, we assumed focus group discussions would be the most beneficial. Online focus groups can be used for potentially sensitive topics [27]. We therefore directed subjects to participate anonymously, without a camera on, via Zoom.

The following research questions were examined from the views of PHNs in public maternity clinics.

- How does screening for IPV occur?
- How do PHNs experience screening for IPV?
- What are the facilitators and barriers to screening for IPV?

### 2.2 Setting and Participants

The study was conducted in public maternity clinics, and the participants were PHNs working in these clinics. Approximately 99.7% of pregnant women in Finland use free public maternity clinic services. The task of the Finnish maternity clinic is to identify problems and disturbances during pregnancy as early as possible and provide guidance in the care of the child. Finnish maternity clinics have been found to narrow health inequalities and prevent exclusion [28].

In the Finnish system, there are typically 8–9 maternity clinic visits during pregnancy. Two of these are examinations with a medical doctor, and the other visits are with PHNs, focusing on monitoring the well‐being of the mother and the fetus. In addition, families are offered 1–2 home visits after the child is born [28]. Guidelines dictate that IPV is systematically screened for in maternity clinics during pregnancy between gestation weeks 22 and 24 using the Domestic Violence Inquiry and Assessment [29]. In Finland, PHNs are educated at a university of applied sciences [30], and they deliver preventive health services for all ages, including pregnant mothers [31]. The eligibility criteria for participation of PHNs were that they (1) were able to participate in the study in Finnish and (2) were regularly meeting with pregnant women in maternity clinics.

### 2.3 Recruitment and Data Collection

Purposive sampling was used to recruit PHNs from three healthcare organisations around Finland. One healthcare organisation could include several municipalities, and the population per municipality varied between 4000 and 90,000 inhabitants. The researcher first approached a contact person for each organisation, who then relayed the research information to potential participants. After receiving the study information, interested PHNs contacted the researcher by email and were assigned to one of four online focus groups. All PHNs (*n* = 12) willing to participate were included, and four focus groups were formed. The researcher did not know the participants, and vice versa, and the researcher had no previous contact with the participating organisations. All participants were contacted by e‐mail to schedule a discussion time. They were also offered the opportunity to contact the researcher by phone or e‐mail before the discussion.

Data were collected in September 2021 via focus group discussions (*n* = 4), administered by the first author (Elina Lähteenmäki), a female with an education in healthcare and clinical experience conducting interviews. The interview questions were developed based on the research questions, national IPV screening guidelines [28], and previous research on IPV during pregnancy and in maternity care [2, 15, 24]. The interview format allowed the possibility for the moderator to ask clarifying and follow‐up questions, depending on the flow of the conversation. No pilot study was conducted. The discussion questions had been emailed to the participants beforehand. Each group comprised two to five participants, and the discussions were conducted remotely via Zoom. Respondents participated from their workplace during the workday, and they were instructed to participate without anyone else present during the discussion. The discussions lasted a total of 231 min (range 50–70 min each). Participants could share their views and understandings in the group, discuss issues, and challenge others to reconsider or change their ideas [32]. As the Zoom discussions were audio‐only, the researcher recorded the interviews and took notes to capture the content and the group dynamics through the participants' verbal responses. All 12 PHNs who volunteered were included in the study. After four focus group discussions, recurring themes indicated that thematic saturation was reached [33], and thus no more recruitment was needed.

### 2.4 Data Analysis

Data analysis followed a constructivist epistemology, acknowledging that knowledge arises from participants' subjective experiences. The first author's professional background in healthcare and maternity services was considered throughout the inductive analysis to minimise bias. No specific preconceptions were applied in advance; instead, an open and data‐driven approach guided the analysis. Coding and category development were discussed collaboratively within the research team to enhance reflexivity and credibility. The data were analysed by inductive content analysis [34] in five phases.

1. The researcher transcribed the discussions verbatim in Word and assigned the participants ID numbers (1–12).
2. Only manifest content was analysed.
3. The original expressions were condensed into meaning units that captured the core content in a more concise form.
4. The condensed meaning units were assigned codes to represent the core content.
5. The codes were then grouped based on similarity into subcategories, which were further abstracted into broader main categories, guided by the research questions.

Coding and category development were discussed and refined collaboratively within the research team to enhance reflexivity and credibility. The PHNs who participated in the focus groups did not take part in reviewing the transcripts or analysis results.

Table 1 illustrates how the data were converted from original expressions to condensed meaning units, codes, subcategories, and finally to main categories. The consolidated criteria for reporting qualitative research (COREQ) [35] were used to guide the reporting of the study.

**TABLE 1:** Example of meaning units and codes in the data analysis process.

Original expressions	Condensed meaning unit	Code	Subcategory	Main category
If both parents are there, then I won't ask. ID1	If both parents are there, then I won't ask	You don't ask about IPV when both parents are present	Asking both parents separately	Organisational protocols
I have done it so that we have this kind of screening form for IPV in the maternity and child health clinic. And we have the recommendation in the maternity clinic's frame program that we ask this, usually in the early stages of screening for this. ID 10	We have a screening form for domestic violence at the maternity and child health clinic, and it is in the maternity clinic's frame programme, even in the early stages of screening	Using the form of the Finnish National Institute for Health and Welfare, in accordance with the recommendations	Using a screening tool	
Personally, I agree that not all cases will come up. ID3	I agree that certainly not all cases will come up	It is suspected that not all cases came to light	Clients rarely report IPV at maternity clinics	It is hard to get a truthful answer
I'm pretty inauthentic, if the other one is like that, you can't say hey you're lying. ID2	I'm pretty inauthentic, you can't say hey you're lying	It is suspected that the client does not respond truthfully	Clients may not answer questions truthfully	

### 2.5 Ethical Considerations

The Finnish guidelines of the Research Ethical Advisory Board were followed [36]. The participants received written and verbal information about the study's aim and procedures. We audio recorded informed verbal consent at the start of data collection. Confidentiality was ensured throughout the study, and data access was restricted to the research team. The study was not estimated to involve any specific ethical risks. However, as IPV is a sensitive topic and participants could have been seen as vulnerable, they were asked to speak without identifying individual cases.

## 3 Results

A total of 12 PHN participated in focus groups. Their median working in maternity clinics was 9.5 years (range 2–35 years). The setting encouraged active and reflective discussion, with participants responding to each other by agreeing, elaborating, or offering different perspectives. This exchange enriched the data and supported the identification of shared themes.

A summary of the main categories and subcategories of the responses to the research questions is presented in Table 2.

**TABLE 2:** Summary of the subcategories and main categories of the research questions.

Subcategories	Main categories	Research question
Using a screening tool Asking both parents separately In accordance with the instructions Using interpreters Trusting intuition Asking again if needed Asking both parents at the same time Explaining what IPV is	Organisational protocols Professional discretion	How does screening for IPV occur?
It's easy to talk about It's hard to talk about It's part of the job description Must ask straight Addressing will prompt a thought about IPV Clients rarely report IPV at maternity clinics Clients do not answer the questions truthfully It isn't always the man The assumption of a family situation	IPV is asked about It is hard to get a truthful answer Preconceived assumptions must be forgotten	Experiences of PHNs when screening for IPV
It's part of the protocol It is done for everyone Existing guidance When client is present alone Clients speak openly Good dialogue Good client relationship Work experience brings certainty Clear treatment path in the organisation Opportunity to get an education Support from colleagues and other professionals	Existing guidance Confidential relationship Work experience Support from the organisation	Facilitators of screening for IPV
You can't ask if both parents are always there Interpreter communication A lot of things to go through Time resource Changing employees There's no confidential relationship with a client A personal relationship with a client The client won't accept any help Fear for the client's safety Fear for your own safety A foreign culture	The conflict between instructions and practice Organisational challenges Challenges in client relationships Cultural differences	Barriers to screening for IPV

### 3.1 How Does Screening for IPV Occur?

Screening for IPV was reported to occur either *through organisational protocols* or *professional discretion*. In line with national guidelines, PHNs generally aimed to ask about IPV when the woman was alone. However, some participants described situations in which they used discretion and were able to address the topic even when both parents were present, either verbally or by utilising a screening form.

If the partner is always present, they will guide the conversation immediately. Then it'll be hard to talk about it anymore. Of course, even the expecting mother can say straight away that they do not want to talk about this issue. ID 4

I can bring it up anytime if I get a gut feeling during the appointment. Even if both parents are present. ID 10

With women who speak foreign languages, IPV is usually asked about through an interpreter because screening forms are not available in all the needed languages. PHNs said they try to reserve a female interpreter for this situation. However, since there is a lot to get through when using an interpreter, experiences of violence may not be asked about.

We'll ask through an interpreter unless they speak English or another language I speak. But the forms, I don't think we have any forms other than in Finnish or English. ID 7

The PHNs emphasised that timing and their own discretion strongly influenced whether screening occurred. Sometimes the timing is not right, for example, when a woman is leaving the clinic, or it seems that the woman does not want to discuss the topic, which is not conducive to initiating discussions about IPV. PHNs reported that, in those situations, IPV screening must be postponed.

And to point out to the women that there are many forms of violence. That it's not just hitting. The client may not always think it's violence. It's not going to affect me. He doesn't hit, he doesn't drink. ID 10

Based on the intuition and professional judgement of PHNs, a woman might be asked again if her initial response lingered in the PHN's mind or evoked concern. Some PHNs pointed out that they were willing to raise the issue when the partner was present. They also questioned the reliability of the responses when both parents filled in the form together.

You wonder how honestly the form is filled out when both parents are there together. ID 10

### 3.2 Experiences of PHNs When Screening for IPV

PHNs described their experiences with IPV screening in terms of: *IPV is asked about*, getting truthful answers can be difficult, and *preconceived assumptions must be forgotten*.

*IPV is asked about*. Screening for IPV is not always easy in maternity clinics, but it is carried out. The PHNs emphasised the value of asking all clients about IPV, as simply raising the topic may encourage reflection, even if the woman does not immediately wish to talk about it.

Another experience was that sometimes *it is hard to get truthful answers*. Most PHNs pointed out that they did not believe that women report IPV in the maternity clinic, even if it is asked about directly. In such situations, IPV was asked about again later, and the client was at least told what IPV is.

I think about that as a percentage, if you think about it, always in these trainings [we hear] how much IPV occurs. So I sometimes think that [in reality] it doesn't come up as often in those surveys, so I get the feeling that I'm sure that a lot of people experience those things. ID 8

*The PHNs felt that preconceived assumptions must be forgotten*. The participants reflected on the importance of avoiding assumptions about families. Some noted that familiarity with a family over multiple pregnancies could lead to a false sense of security, which could result in overlooking the need to ask about IPV. The PHNs also highlighted the need to avoid assuming that the perpetrator is always the male partner.

It could just as well be the father experiencing violence—we shouldn't assume. ID 3

### 3.3 Facilitators for Screening for IPV in Maternity Clinics

Facilitators of screening could be divided into four categories: *the existing guidance*, *a confidential client relationship*, *work experience*, and *organisational support*.

*Existing guidance* made screening for IPV easier. Systematically asking each family about IPV made the screening easier. If clients are assured that everyone is asked about IPV, PHNs do not have to worry that a client will feel stigmatised. Every organisation represented was reported to have screening methods, and these were used to varying degrees. Screening tools were also seen as a facilitating factor and helpful when the family was unfamiliar to the PHN.

It's so like easy to take that form and go through it based on it. If there's anything. And anyway, talk about IPV. That form is easy and good. ID 5

In contrast, when working with familiar families, PHNs reported that they sometimes preferred to ask about IPV directly without using the screening form.

Another facilitator was *a confidential client relationship*. Several PHNs held a combined role of a maternity and child health clinic worker, which allowed them to build familiarity with families over time. The participants also reflected that, when clients were open about their lives and communicated effectively, it was easier to address IPV.

We may have discussed other difficult issues, so yes, it often makes it easier to talk about it, including this topic. ID 8

*Work experience* was also found to be a facilitator for screening for IPV. Some PHNs stated that asking about IPV became easier over time, while others felt it remained challenging.

This is something you would always want to reinforce with your own expertise. To be able to recognize and help families, to have courage [myself]. And as my career gets longer, that's what I'm looking forward to, and I'm sure it'll get easier every time. ID 2

*Support from their organisation* was described as a key facilitator for IPV screening. PHNs emphasised that such support helped them know how to guide a family forward after a positive disclosure. Clear care pathways, collegial support, training opportunities, and a shared sense of responsibility further supported their ability to address IPV. However, educational background and the perceived usefulness of available materials varied between participants.

It helps that we ask the same from everyone, so I can explain it's not personal. ID‐1

### 3.4 Barriers to Screening for IPV in a Maternity Clinic

Barriers to IPV screening were divided into five categories: *conflict between instructions and practice*, *language barriers*, *organisational challenges*, *challenges in client relationships*, and *cultural differences*.

*Conflict between instructions and practice* when screening for IPV was evident when clients never visited the maternity clinic alone and PHNs did not have the opportunity to screen for IPV in private, as instructed.

If both are always there, it might not get asked. ID 5

Another common barrier was the lack of a common language. Although the goal is always to get a female interpreter, some foreign language‐speaking women do not want to talk about their home situation or relationship with an interpreter.

I do see it as a challenge to bring up this issue of IPV, especially if there is no common language. And they may not be able to speak English or the kind of languages I speak now, and it is a challenge, especially through telephone interpreting, so it is very, very little what I take, especially if you have a male interpreter and a female client here, then it is quite a bit of a thing. ‐ID 9

*Organisational challenges* include the lack of time or uncertainty about how to proceed if IPV is disclosed. Families can have a variety of needs, and many aspects are included in maternity clinic visits. There is a lack of time to cover everything in one visit. This especially concerns families with multiple difficulties or issues. PHNs also expressed that screening for IPV when several other difficulties had already been addressed during the visit felt unreasonable.

Whenever you ask about it, you have to assume that if she says yes from there, then you don't have time for me to stop and discuss it from an ethical point of view. So it's terrible to ask a client how you're doing if you don't have a long time to go [and you have] to stop at what they might open there. ID 8

*Challenges in client relationships* may prevent screening for IPV, and clients simply may not want to accept help or talk about it. Some PHNs worked in a relatively small locality and found it challenging if the family was familiar with their personal life. If they dared to ask, they could not be sure whether the client would answer truthfully in these situations. The lack of a confidential client relationship also posed challenges.

If you really knew this client…. Would it then feel like a bit of prying around at that point even it would be professional, it could be difficult… But, of course, I'd have to ask. ID 6

*Cultural differences* posed another significant challenge. In some cultures, the whole family typically participates in the clinic visit, making it difficult to talk with the pregnant woman alone. IPV can also be seen differently depending on the culture, and it may be difficult for the client to share certain information with a stranger.

In my opinion, these are going to be great challenges for these immigrant families. How you bring it up with them, and what their culture is. Many times, in these families there is that father involved in the visits, and that is what it is really difficult to bring up through an interpreter, as it is not simple. ID 3

## 4 Discussion

The purpose of this study was to explore PHNs' views on screening for IPV in public maternity clinics. We found that the PHNs' awareness of their important role at the maternity clinic was a facilitating factor for screening for IPV. This may have further facilitated them performing screening as advised. This factor is possibly connected to Finnish national guidelines stipulating systematic screening methods for IPV [37]. PHNs at maternity clinics can offer both psychoeducation and forward guidance if necessary to parents [29].

Organisations have tools for screening IPV and various methods for meeting with pregnant women or their partners one‐on‐one to encourage more open communication. However, these tools are not standardised. Additionally, the Domestic Violence Inquiry and Assessment by the Finnish Institute for Health and Welfare is used between gestation weeks 22–24. Previous studies have shown a transfer of responsibility, the wish for another professional to bring up the topic [24], or the idea that there is no obligation to ask about IPV [25]. However, in this study, PHNs described that they did not transfer this responsibility to a colleague or another professional group nor did they assume that they did not need to screen for IPV. In some situations, though, support from a medical doctor—in screening or in providing forward guidance—was needed. Other studies have found that midwives might assume that pregnant women who have experienced relationship violence will bring it up themselves [25]. Being aware of how many people are affected by IPV could increase the understanding of the importance of screening. In Finland, the work of a PHN is independent and preventive. This may be why PHNs in this study did not report transferring responsibility to another professional.

In this study, as in previous studies, participants identified a lack of privacy as one of the most common barriers to screening for IPV [38]. Here, the participants described that one possible way to ensure privacy was to invite the pregnant woman or the partner to the maternity clinic alone. In some organisations, pregnant women were invited to visit one time alone during pregnancy, and some organisations also arranged a visit for the partner alone during the pregnancy. The respondents had also used e‐forms to be completed before the maternity clinic visit. This allowed a pregnant woman to fill out the form independently, which saved time at the maternity clinic. The usefulness of e‐forms in screening for IPV should be studied. Currently, the e‐forms used by organisations participating in the study were created by the organisations themselves.

The PHNs generally viewed IPV screening as a standard part of their work, but they also recognised several barriers that sometimes prevented them from conducting it. The participants reflected that clients might not always disclose their experiences of IPV, as the number of reported cases appeared lower than what they had expected based on prevalence data [23].

Although the respondents felt they had received sufficient training for screening, one barrier to screening was a lack of time. Further, a shortage of resources, particularly of staff availability, is known to be one of the most common reasons for not screening for IPV [39]. It may be that the lack of resources not only means there is no time to screen; IPV may not be screened for because there is no time to help the client if they report violence. The topic of IPV may remain unbroached, or a truthful answer may not be given if the professional seems busy or uncertain when asking about it. A previous study has shown that asking about violence and offering support can determine which victims choose to share their experiences, and the absence of these factors can prevent victims from reporting [40].

Cultural differences should increasingly be considered as immigration increases. To create a friendly and trusting relationship, a professional interpreter should be involved at every healthcare visit [41]. In this study, the PHNs in Finland said that foreign language‐speaking pregnant women were less often screened for IPV due to the language barrier. At the beginning of 2021, 5.3% of people living in EU‐member states were non‐EU migrants [42]. In Finland, the share of speakers of foreign languages in the total population is 9% [43]. Currently, there is a lack of research on interventions for pregnant immigrants to address IPV [44]. Immigrant women may experience forms of IPV that are very different from those PHNs are used to, and violence may not be recognised. IPV can manifest as financial control, for example, leaving a woman to have to survive completely without money [45]. In some cultural contexts, IPV may take less visible forms, such as social isolation, restriction of movement, or control over access to healthcare services [46]. Such forms of violence may be harder for PHNs to recognise, highlighting the need for training in cultural competence and culturally specific signs of IPV. To meet the different needs of immigrant women, culturally competent cooperation between various agencies is needed [41]. Within the context of supporting immigrant families, promoting gender equality in families from conflict zones may support women's ability to recognise and disclose IPV; it may also help professionals challenge cultural norms that normalise control or violence [47]. Studies show a need for healthcare organisations to promote gender equality for families entering the country from conflict zones and to question attitudes and practices that allow men to be violent in relationships. This may be relevant in the context of immigrant families, where underlying norms can influence both women's willingness to disclose and professionals' readiness to ask [47].

Systematic screening for IPV is necessary for all risk groups, including pregnant women [46]. However, this study found that PHNs felt that IPV is not reported often, even when routine screening is conducted. Further research could help provide an understanding of what factors influence a pregnant woman's decision to report IPV to professionals. Most importantly, more consideration should be given to how victims of IPV could share their experiences and receive help. This is not only a task for public healthcare; other authorities such as social workers need to actively provide support and raise the issue of partner violence when necessary [48].

## 5 Strengths and Limitations

Some limitations should be considered when interpreting the study's findings. The data were collected and analysed by one researcher, but the subcategories and categories were reviewed and confirmed with the research team. The research context differs from that of other countries. Finnish maternity clinics are unique: they are free for all pregnant women and governed by national guidelines [28]. It is possible that the selected PHNs were already interested in the subject and therefore motivated to screen for IPV. If so, a distorted picture of the state of IPV screening in Finland may have been formed.

A strength of this study is that the participants were willing to share their experiences despite the sensitivity of the subject, which enabled new data to be obtained. We sent the discussion questions to the participants beforehand to allow sufficient time to consider their participation and to be prepared for the discussion. We emphasised the voluntary nature of participation to further support participants' willingness to share their thoughts and experiences. They could refuse or withdraw from the study at any time. Before and during the focus group discussions, we emphasised the confidentiality and anonymity to enable an open dialogue. This is especially important considering increasing evidence about the occurrence of IPV [1].

Another strength is that the coding and category development were reviewed and confirmed in collaboration with the research team, which increased the trustworthiness of the analysis. The study method was chosen to produce deeper information rather than generalising information. Efforts were made to analyse and report the material accurately, with support from quotations from the material to improve credibility. Moreover, to support the study's transferability, we included participants from various experiences and geographical locations.

## 6 Conclusions

This study shows that the PHNs perceived that Finnish maternity clinics have a functioning approach to IPV screening. This study suggests that systematic screening for IPV was described as a part of standard care. This is supported by national coherent guidance on screening. However, the findings indicate that, despite systematic screening, supportive questionnaires, and national guidelines, it remains difficult to accurately identify IPV in maternity care settings. It would be beneficial to explore alternative approaches to discussing IPV with clients and recognise that disclosure is ultimately the client's decision. As immigration increases, more attention should be paid to linguistic and cultural diversity in maternity care. To support effective screening, PHNs would benefit from additional training in culturally sensitive communication and in recognising less visible forms of IPV.

## Author Contributions

E.L. conceptualization, Project administration, Investigation, Data curation, Formal analysis, Writing – original draft, Writing – review and editing. H.L.‐K. conceptualization, Project administration, Data curation, Formal analysis, Writing – original draft, Writing – review and editing. K.M. conceptualization, Project administration, Data curation, Formal analysis, Writing – original draft, Writing – review and editing.

## Ethics Statement

The study design was approved by the ethical committee of the University of Turku (8/2021). We obtained permission from each study site.

## Conflicts of Interest

The authors declare no conflicts of interest.

## Data Availability

The data that support the findings of this study are available on request from the corresponding author. The data are not publicly available due to privacy or ethical restrictions.
